# Monofluorophos–Metal Complexes: Ripe for Future Discoveries in Homogeneous Catalysis

**DOI:** 10.3390/molecules29102368

**Published:** 2024-05-17

**Authors:** Alexandra M. Miles-Hobbs, Paul G. Pringle, J. Derek Woollins, Daniel Good

**Affiliations:** 1 The School of Chemistry, University of Bristol, Cantock’s Close, Bristol BS8 1TS, UK; 2 Department of Chemistry Khalifa University, Abu Dhabi P.O. Box 127788, United Arab Emirates; jdw3@st-andrews.ac.uk

**Keywords:** fluorophosphites, fluorophosphines, coordination chemistry, homogeneous catalysis

## Abstract

The discovery that cyclic (ArO)_2_PF can support Rh-catalysts for hydroformylation with significant advantages in tuning regioselectivity transformed the study of metal complexes of monofluorophos ligands from one of primarily academic interest to one with potentially important applications in catalysis. In this review, the syntheses of monofluorophosphites, (RO)_2_PF, and monofluorophosphines, R_2_PF, are discussed and the factors that control the kinetic stability of these ligands to hydrolysis and disproportionation are set out. A survey of the coordination chemistry of these two classes of monofluorophos ligands with d-block metals is presented, emphasising the bonding of the fluorophos to d-block metals, predominantly in low oxidation states. The application of monofluorophos ligands in homogeneous catalysis (especially hydroformylation and hydrocyanation) is discussed, and it is argued that there is great potential for monofluorophos complexes in future catalytic applications.

## 1. Introduction

**Figure 1:**
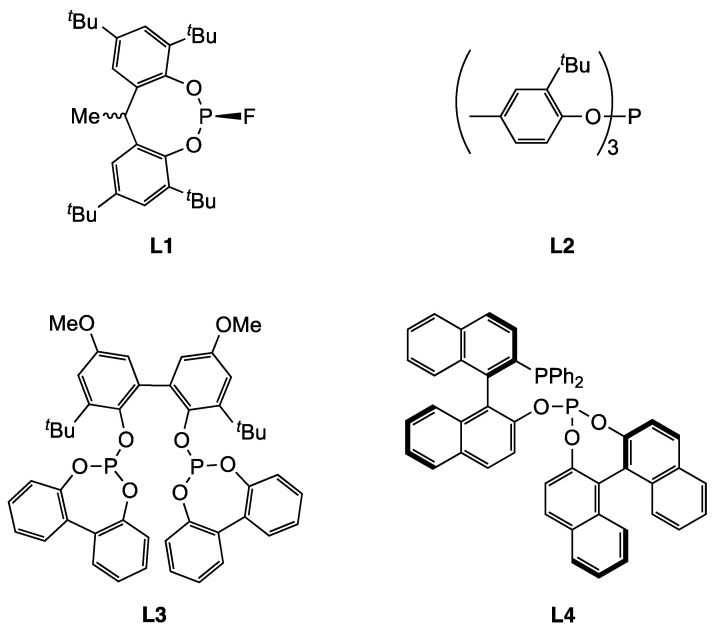
Ethanox-398 (**L1**) and some other landmark phosphite ligands **L2**–**L4** for Rh-catalysed hydroformylation.

Phosphorus ligands containing P–C, P–N, and P–O bonds are ubiquitous in homogeneous catalysis. By contrast, fluorophos ligands (those containing a P–F bond) have attracted relatively little attention in catalysis, despite the extensive fluorophos coordination chemistry of late transition metals that has been developed and the industrial interest in the application of monofluorophosphite **L1** (Figure 1) in Rh-catalysed hydroformylation dating back to 1998 [1]. In other contexts, **L1** (commercial name: Ethanox 398) has been employed as an antioxidant [2] and as a flame retardant [3].

The extreme electronegativity of fluorine means that it can withdraw electron density from any atom it is bonded to, contributing to its reputation as the *Tyrannosaurus Rex* of chemistry [4]. It should be noted that the electron-withdrawing power of F is a σ-inductive effect and, in some cases, this is offset by an electron-donating π-resonance effect (see later) [5]. This property, combined with the diminutive size of P–F (only P–H is smaller), makes the steric and electronic properties of an F substituent of particular academic interest. The high electronegativity of F would be expected to enhance the π-acceptor capacity of ligands containing P–F bonds compared to analogous ligands containing P–O bonds. Since one of the reasons cited for the success of phosphites such as **L2–4** (Figure 1) as ligands in Rh-catalysed hydroformylation is their strong π-acceptor capacity, it is understandable why monofluorophosphite **L1** performs well in hydroformylation [6,7,8,9,10,11].

The simplest fluorophos ligand, PF_3_, has a special place in coordination and organometallic chemistry as a ligand that has π-acceptor properties on par with, or surpassing, those of CO [12]. The volatility of some PF_3_ complexes has made them attractive for applications in chemical vapour deposition [13,14,15] and recently, a PF_3_ complex, identified as [Co_2_(μ-CO)_2_(CO)_2_(PF_3_)_4_], was reported to be a catalyst precursor for 1-hexene hydroformylation [16]. However, progress in the application of PF_3_ as an ancillary ligand is hampered by it being an odourless gas with toxicity similar to phosgene [17], and it is not amenable to chemical modification.

**Figure 2:**
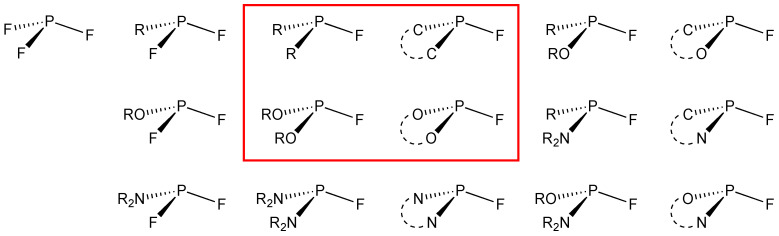
A selection of P–F containing monophos ligands (R = alkyl or aryl group) including P-heterocycles showing the diversity of ligands that are potentially available. The structures in the red box are the subject of this review.

There are no such disadvantages for the collage of P–F ligands, depicted in Figure 2, which have C-, O-, or N-substituents. These substituted fluorophos ligands have the advantages of being systematically modifiable via R substituents and they are generally straightforward to synthesise.

The focus of this review is acyclic and cyclic monofluorophos ligands of the type (RO)_2_P–F and R_2_P–F, since these are amongst the simplest achiral P^III^ compounds that contain a P–F bond. Both of these classes of P-ligand have attracted considerable academic and industrial interest since the 1960s, including in the area of homogeneous catalysis. To the best of our knowledge, there has not previously been a review of monofluorophos ligands, although difluorophos ligands have been reviewed [18]. The topics covered in this review include (1) the synthetic routes to monofluorophosphites and monofluorophosphines; (2) the factors controlling the stability of monofluorophos ligands that limit their applications; (3) the transition metal coordination chemistry of monofluorophos ligands that may be pertinent to an understanding of their role in homogeneous catalysis; (4) the homogeneous catalysis that has been reported with metal–monofluorophos complexes. This review is not comprehensive and there is a bias to more recent developments that build upon the early foundational work reported by the groups of Schmutzler and Nixon. The main conclusion that is drawn from this review is that the tunability of the steric and electronic effects in monofluorophosphites and monofluorophosphines augurs well for future applications of these and related classes of P–F ligands in homogeneous catalysis.

## 2. Monofluorophosphites

### 2.1. Synthesis and Hydrolytic Stability of Monofluorophosphites

**Scheme 1:**
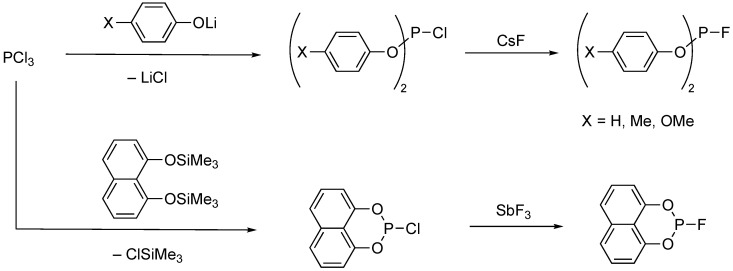
Typical examples of the synthesis of acyclic and cyclic monofluorophosphites.

Cyclic and acyclic monofluorophosphites are most readily prepared from the corresponding chlorophosphite, PCl(OR)_2_, and a source of fluoride, such as CsF or SbF_3_. The precursor chlorophosphites are prepared from PCl_3_ and the appropriate phenol/alcohol, or a siloxy derivative (as exemplified in Scheme 1) [19]. Monofluorophosphites have also been made from PCl_2_F, but this precursor is not readily accessible [20,21].

At first sight, the prospects for using any halophos ligand of the type Z_2_P–Hal (Z = alkyl, aryl, OR, OAr, NR_2_; Hal = F, Cl, Br, I) in catalysis may appear bleak because of the reactivity of P-Hal bonds. For example, chlorophos compounds (Z_2_P–Cl) are normally viewed as useful intermediates rather than ligands because they react readily with a wide range of C-, O-, or N-nucleophiles [22]; this reactivity makes chlorophos ligands incompatible with many reactive functional groups. Moreover, chlorophos compounds commonly fume in air because of their high susceptibility to hydrolysis, during which HCl is produced (Equation (1), X = Cl).



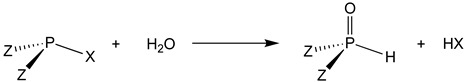

(1)


The favourable thermodynamics of P–Cl hydrolysis are largely driven by the P=O bond formation in the P-containing product (Equation (1), X = Cl). However, the thermodynamics of P–OAr hydrolysis (Equation (1), X = OAr) are at least as favourable as those of P-Cl hydrolysis and yet ligands containing P-OAr groups are widely used in coordination chemistry and catalysis. It can therefore be surmised that the high reactivity of chlorophosphites is primarily due to their high kinetic lability. Indeed, chlorophosphites that are remarkably stable to moisture have also been developed and some have been applied in catalysis [23,24].

It has been shown that phosphite P–O bonds can be stabilised to hydrolysis by integrating them into cyclic structures and/or incorporating bulky hydrophobic groups into the ligand framework, as in aryl phosphite ligands **L2–4** (Figure 1). Indeed, diphosphite **L3** and its derivatives have been successfully applied in large scale industrial hydroformylation processes [7]. It is of no surprise, therefore, that the Eastman monofluorophos ligand **L1** is a phosphadioxacycle which contains bulky *t*-butyl substituents that shroud the P–F moiety [1].

**Figure 3:**
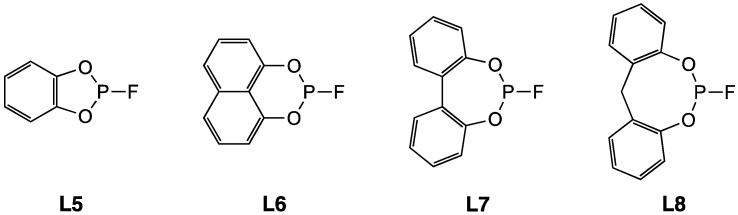
Cyclic monofluorophosphites **L5**–**L8** with ring sizes of 5–8, respectively.

While **L1** is reportedly stable to hydrolysis [25], the hydrolytic stability of the related cyclic monofluorophosphites **L5–8** (Figure 3) in aqueous methanol depends on ring size: the half-lives increase in the order **L5** < **L7** ~ **L8** < **L6** [19].

### 2.2. Coordination Chemistry of Monofluorophosphites

**Scheme 2:**
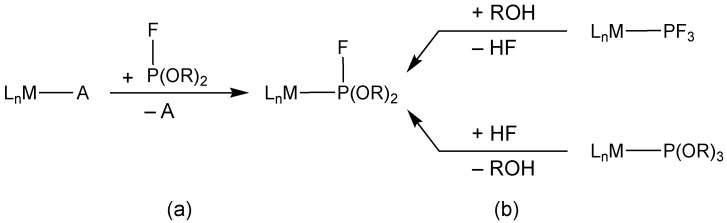
Routes to monofluorophosphite complexes: (**a**) conventional substitution at the metal of neutral ligand A by monofluorophos ligand; (**b**) substitution at the phosphorus of a coordinated P-ligand.

Metal complexes of monofluorophosphites have been produced by the two routes shown in Scheme 2: (a) by substitution of a labile, neutral ligand (A) by a monofluorophosphite; (b) by methanolysis of a coordinated PF_3_ or by addition of an equivalent of HF to a coordinated P(OR)_3_.

#### 2.2.1. Group 6 Metal Complexes of Monofluorophosphites

**Scheme 3:**
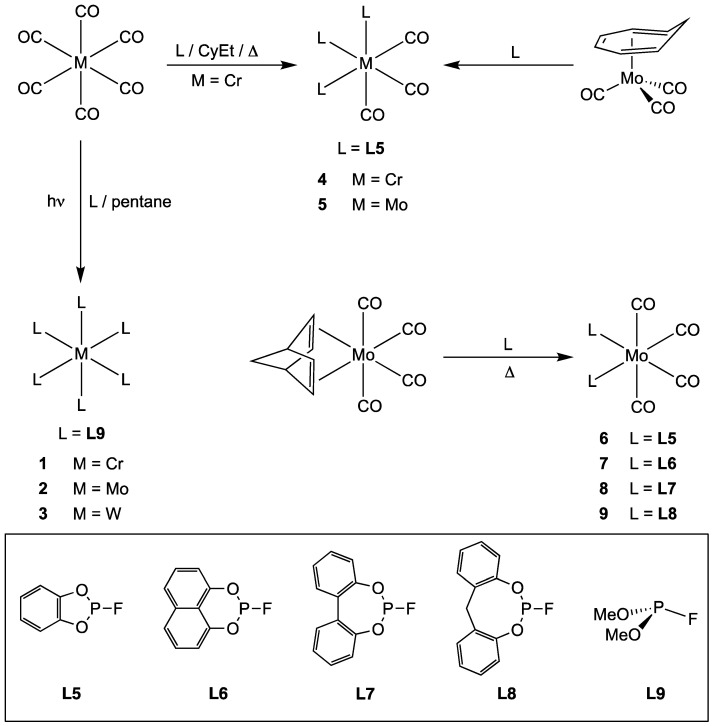
Group 6 metal complexes of monofluorophosphites.

The range of Group 6 metal(0) complexes of monofluorophosphites that have been prepared is summarised in Scheme 3 [20,26,27]. UV photolysis of each of the metal hexacarbonyls in the presence of (MeO)_2_PF (**L9**) gave the homoleptic complexes **1–3** [27]. [Cr(CO)_6_] reacts with **L5** to give the trisubstituted **4** while the molybdenum analogue **5** is formed when **L5** reacts with [(cycloheptatriene)Mo(CO)_3_] [26].

The *cis-*disubstituted Mo complexes **6–9** were prepared by substitution of the norbornadiene ligand in [Mo(nbd)(CO)_4_] with the cyclic monofluorophosphites **L5–L8** and the products were fully characterised, including by X-ray crystallography. The IR data for **6–9** are consistent with the π-acceptor capacities of **L5–L8** lying between those of PF_3_ and P(OPh)_3_. The ν_CO_ values for the highest frequency band increases in the order P(OPh)_3_ < **L8** ~ **L7** < **L6** < **L5** < PF_3_, which is consistent with the π-acceptor capacity of the cyclic phosphites increasing as the ring size decreases [19].

#### 2.2.2. Group 8 Metal Complexes of Monofluorophosphites

**Scheme 4:**
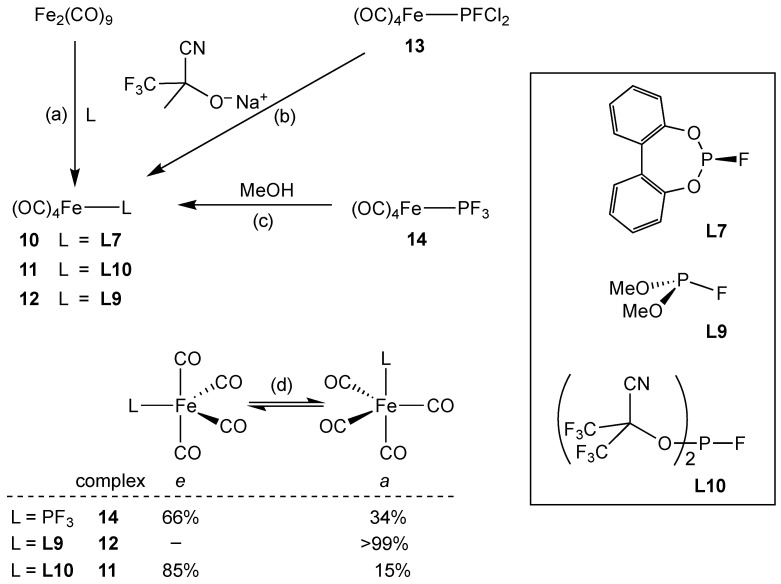
Iron(0) complexes of monofluorophosphites. Routes (a)-(c) and equilibrium (d) are referred to in the text.

The synthesis of the iron(0)–monofluorophosphite complexes **10–12** is summarised in Scheme 4. Complex **10** is formed by addition of ligand **L7** to [Fe_2_(CO)_9_] (Scheme 4, route (a)) [20]. Complex **11** is produced by two routes: (1) addition of ligand **L10** to [Fe_2_(CO)_9_] (Scheme 4, route (a)); (2) treatment of the Fe–PFCl_2_ precursor complex **13** with the sodium alkoxide nucleophile shown in Scheme 4 route (b) [28].

Complex **12** has been identified by IR spectroscopy as a product of the methanolysis of the PF_3_ complex **14** in a detailed study of the alcoholysis of [Fe(PF_3_)_x_(CO)_5-x_] (x = 1–4) species [29].

The equilibrium proportions of equatorial (*e*) and apical (*a*) isomers of [Fe(CO)_4_L] can be determined by IR spectroscopy; sterically demanding and good π-acceptor ligands prefer to bind at the equatorial sites [29]. As shown in Scheme 4 (d), for complex **14**, the predominant isomer has the PF_3_ equatorial, although the *e*:*a* ratio is close to the statistical 60:40 ratio, reflecting the similarity of PF_3_ and CO as ligands. For complex **12**, only the apical isomer was detected, consistent with **L9** being small and a poorer π-acceptor than PF_3_. For complex **11**, a higher proportion of equatorial isomer was present than even in the PF_3_ complex **14**, as expected for the bulky **L10**. The ν_CO_ values for the complexes **14** and **11** are very similar, showing that PF_3_ and **L10** have similar π-acceptor properties. This demonstrates that the steric and electronic effects of monofluorophosphite ligands can be controlled via the phosphorus alkoxy substituents.

**Scheme 5:**
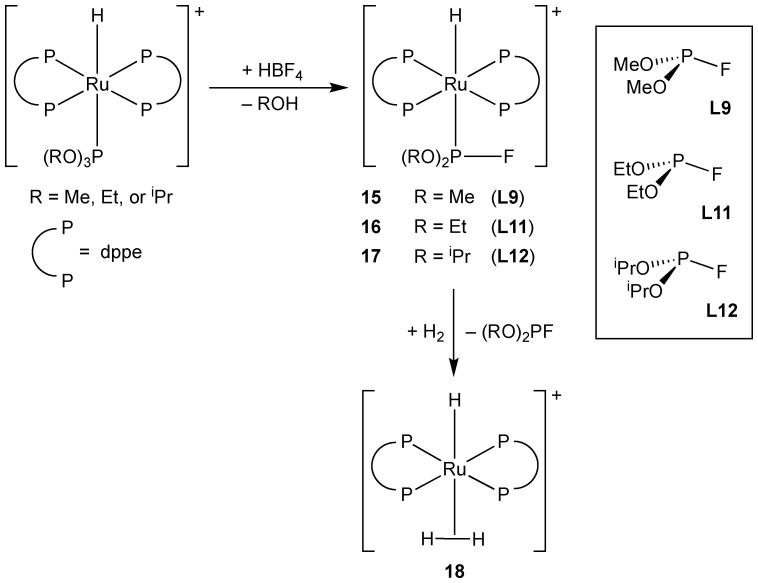
In situ generation of monofluorophosphite ligands on ruthenium(II).

The ruthenium(II) phosphite complexes *trans*-[(dppe)_2_Ru(H){P(OR)_3_}]^+^ react with HBF_4_ to give the homologous series of monofluorophosphite complexes **15–17** (Scheme 5); the HBF_4_ is providing the source of HF in these reactions. The coordinated monofluorophosphite ligands **L9**, **L11**, and **L12** are readily displaced by a H_2_ to give the η^2^-H_2_ complex **18** (Scheme 5) [30].

#### 2.2.3. Group 9 Metal Complexes of Monofluorophosphites

**Scheme 6:**
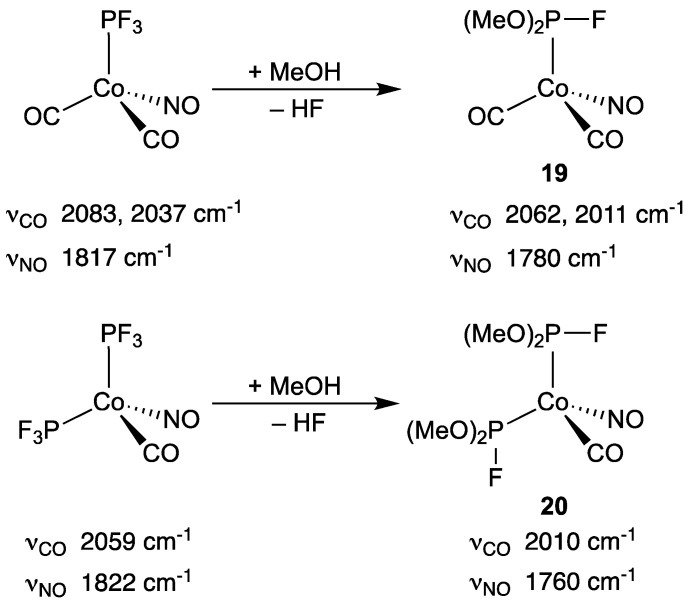
Monofluorophosphite derivatives of nitosylcobalt(−I) complexes.

The tetrahedral cobalt complexes **19** and **20** containing the coordinated **L9** have been separated by preparative GLC from the mixtures obtained by methanolysis of the corresponding PF_3_ complexes (Scheme 6) [31]. The IR spectra of the complexes showed that the ν_CO_ and ν_NO_ stretching bands are both shifted to significantly lower wavenumber in the monofluorophosphite complexes **19** and **20** with respect to their PF_3_ precursors, consistent with **L9** being a poorer π-acceptor ligand than PF_3_.

**Scheme 7:**
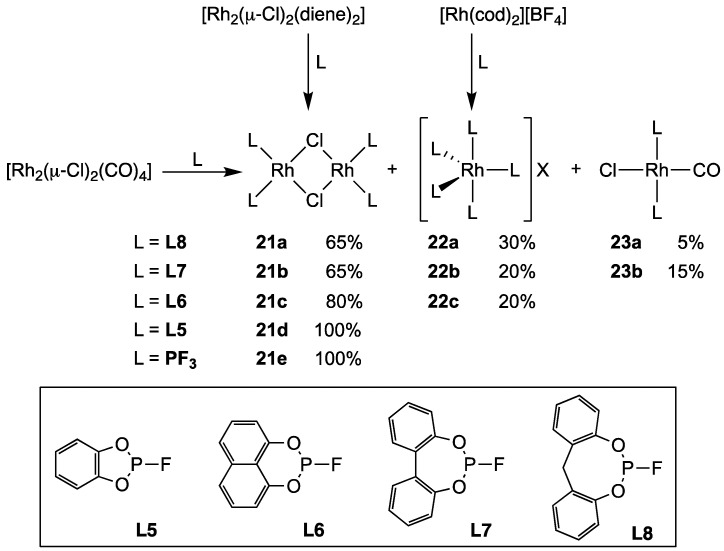
Cyclic monofluorophosphite chemistry of rhodium(I).

The rhodium(I) chemistry with the cyclic monofluorophosphites **L5–L8** is summarised in Scheme 7 [19]. Treatment of [Rh_2_Cl_2_(CO)_4_] with **L5–L8** gave the three products **21–23** in the proportions shown in Scheme 7. These products were characterised by multinuclear NMR spectroscopy and comparison of the spectra with the products exclusively formed from [Rh_2_Cl_2_(diene)_2_] (diene = 1,5-hexadiene or 1,5-cyclooctadiene) and [Rh(cod)_2_][BF_4_]. There is a consistent trend of increasing proportion of binuclear complex **21** formed with decreasing ring size; indeed, with **L5**, binuclear **21d** is exclusively formed. It is significant that PF_3_ is the only other monophos ligand that selectively forms the binuclear product **21e** [32,33]. The interpretation of these observations is that **L5** and PF_3_ are sufficiently good π-acceptors to displace the CO from the Rh.

The trend of increasing PF_3_-like behaviour with decreasing size of phosphacycle in relation to the reactions of **L8–L5** with [Rh_2_Cl_2_(CO)_4_] parallels the trend observed in the spectroscopic properties of *cis*-[Mo(CO)_4_(L)_2_] (see above) [19].

#### 2.2.4. Group 10 Metal Complexes of Monofluorophosphites

**Scheme 8:**
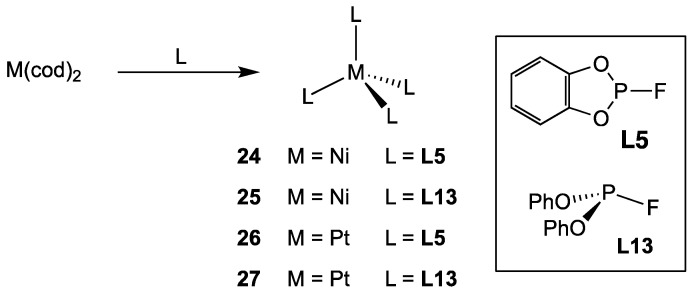
Nickel(0) and platinum(0) chemistry of monofluorophosphites.

The homoleptic nickel(0) and platinum(0) complexes **24–27** containing monofluorophosphites **L5** or **L13** were prepared (Scheme 8) [34,35] and their ^31^P and ^19^F NMR spectra were analysed extensively because they are rare examples of [AX]_4_ spin systems [35,36]. It was noted that the ^2^*J*_P,P_ values for the Ni(0) complexes **24** and **25** (*ca.* 20 Hz) are significantly smaller than for the analogous Pt(0) complexes **26** and **27** (*ca.* 100 Hz), although no rationale was given for this large difference [35]. The nickel(0) complexes **24** and **25** were originally prepared from [Ni(CO)_4_] [26,34] but it was shown that complexes **24–27** can be conveniently prepared from the corresponding [M(cod)_2_] (Scheme 8) [35].

**Scheme 9:**
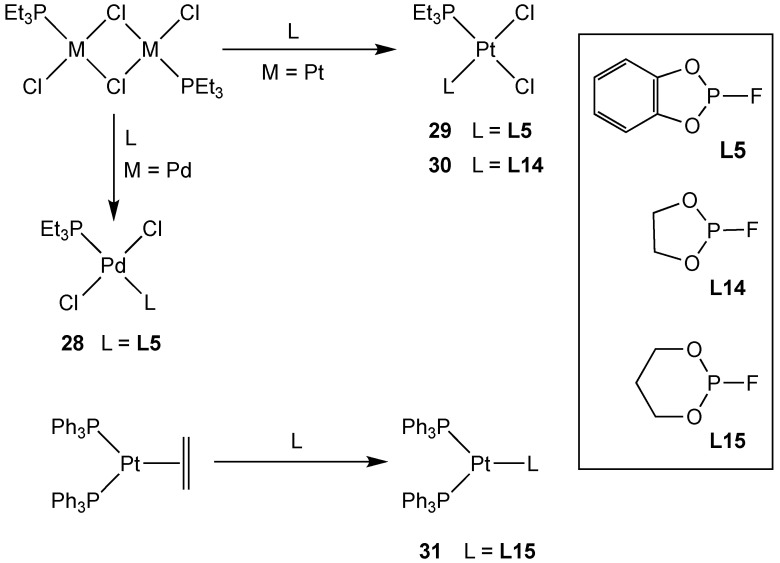
Platinum(II) and palladium(II) chemistry of monofluorophosphites.

The *trans-*palladium(II) and *cis-*platinum(II) complexes **28** and **29**, containing the cyclic monofluorophosphite **L5**, were prepared by cleavage of the corresponding binuclear complex (Scheme 9) [37]. The phosphacycle **L14**, which can be viewed as a saturated analogue of **L5**, forms the *cis*-platinum(II) complex **30**; comparison of the ^31^P NMR parameters for **29** and **30** shows that they are similar, e.g., *J*_Pt,P_ = 5600 and 5490 Hz, respectively. The platinum(0) complex **31** contains monofluorophosphite **L15**, a saturated analogue of **L6** (Scheme 9) [37].

**Scheme 10:**
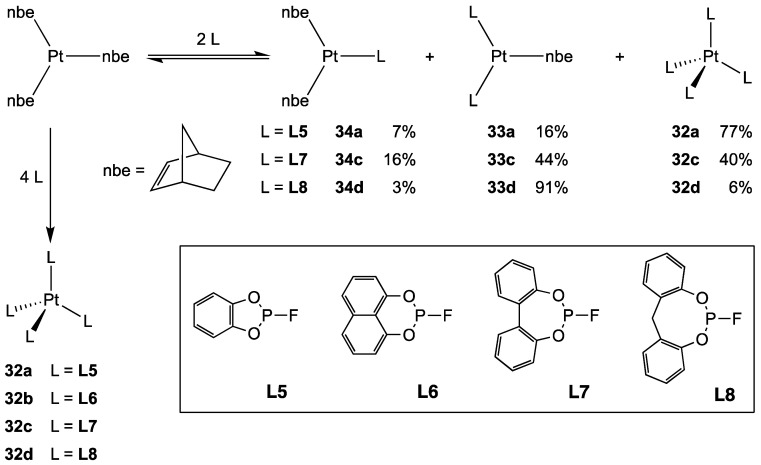
Platinum(0) chemistry of monofluorophosphites.

The tetrahedral platinum(0) complexes **32a–d** are readily formed by the addition of 4 equiv. of **L5–L8** to [Pt(nbe)_3_] (nbe = norbornene). Complex **32b** crystallised from solution even when a sub-stoichiometric amount of **L6** was added (Scheme 10). However, the addition of 2 equiv. of **L5**, **L7** or **L8** to [Pt(nbe)_3_] in THF gave mixtures of [Pt(L)_4_] (**32a,c,d**) [Pt(L)_2_(nbe)] (**33a,c,d**), and [Pt(L)(nbe)_2_] (**34a,c,d**), identified from their characteristic ^31^P and ^195^Pt NMR signals (Scheme 10) [19]. The ratios of complexes observed at equilibrium (Scheme 10) were rationalised to be the result of the competing steric and electronic factors for the nbe and monofluorophosphite ligands; for example, while [Pt(L)_4_] is more sterically crowded than [Pt(L)_2_(nbe)], the greater π-acceptor properties of monofluorophosphites makes them better than norbornene at stabilising Pt(0) [19].

### 2.3. Catalysis with Complexes of Monofluorophosphites

#### 2.3.1. Hydroformylation Catalysis with Rhodium Complexes of Monofluorophosphites

**Scheme 11:**
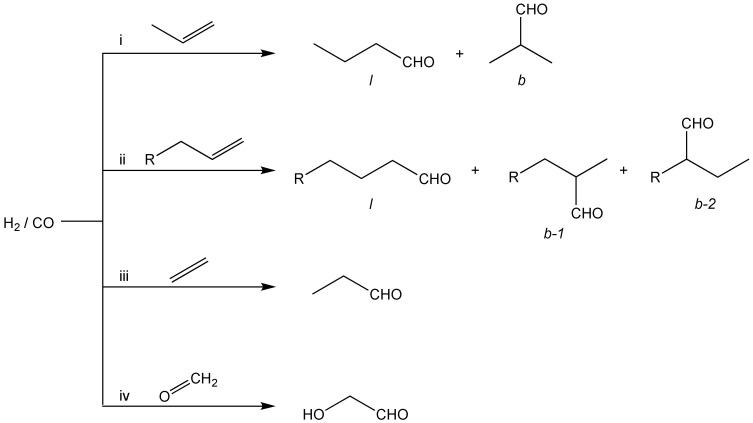
Rh-monofluorophosphite catalysed hydroformylation of alkenes (reactions (**i**–**iii**)) and formaldehyde (reaction (**iv**)).

The most notable example of the application of monofluorophos ligands in homogeneous catalysis is the use of cyclic monofluorophosphites such as **L1** in the Rh-catalysed hydroformylation reactions, reported by Eastman and shown in Scheme 11 [1,25]. Initially, the application of monofluorophosphite ligands in catalysis was approached with scepticism, as it was suspected that monofluorophosphites may be thermally unstable, and be prone to hydrolysis, especially at elevated temperatures, generating hydrogen fluoride (HF), which is a known catalyst poison [25,38,39]. However, it was demonstrated that **L1** is stable to degradation at temperatures up to 350 °C and stable to hydrolysis even in refluxing aqueous isopropanol, with no free fluoride ions detected [40]. While acidic conditions promote the degradation of monofluorophosphites, it has been shown that the catalyst system can be stabilised by the addition of an epoxide or a complex such as [Co(acac)_3_] [41,42].

The striking stability of **L1** is attributed to the 8-membered phosphacycle which entropically stabilises the ligand to P–O cleavage and to the *^t^*Bu substituents which sterically shield the P atom and provide a hydrophobic environment in the vicinity of the P–F bond.

**Figure 4:**
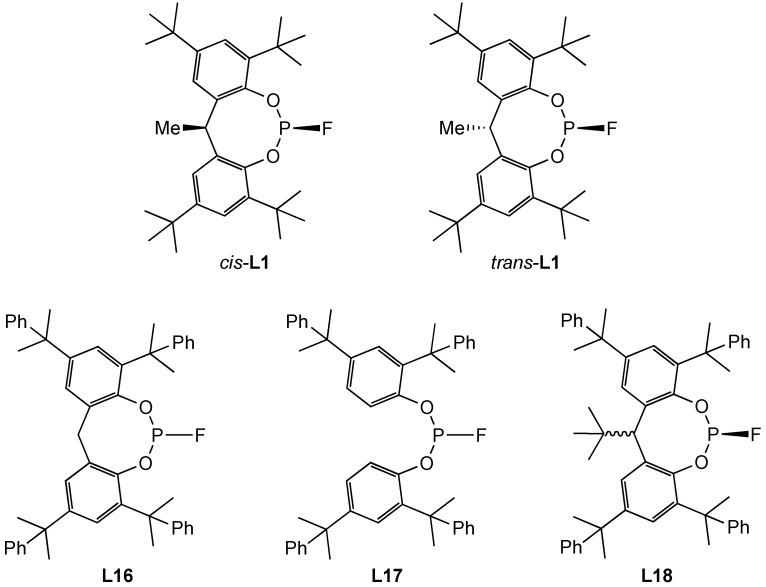
Some of the Eastman fluorophophites used as ligands in hydroformylation.

Ligand **L1** exists as two geometric isomers, labelled *cis-***L1** and *trans-***L1** in Figure 4, associated with the relative stereochemistry of the F substituent on P and the Me substituent on the CH of the ligand backbone. The isomers of **L1** have been separated, and it was shown by ^31^P NMR spectroscopy that, when [Rh(CO)_2_(acac)] was treated with 2 equiv. of *cis*-**L1**, a mono-ligated RhL_1_ species was produced whereas with 2 equiv. of *trans*-**L1** a bis-ligated RhL_2_ species was the product. Furthermore, *trans*-**L1** readily displaced *cis*-**L1** from its Rh(acac) complex, showing that *trans*-**L1** has a greater affinity for the Rh(I) centre than *cis-***L1** [43,44]. These differences in coordination chemistry are likely due to the 8-membered heterocycle having to adopt a more strained ring conformation in *cis*-**L1** than in *trans*-**L1** in order to accommodate the bulky metal moiety being bound at a pseudo-equatorial site. The observed coordination chemistry differences of the isomers of **L1** may be the source of the differences in hydroformylation activity and selectivity that are observed with the various mixtures of isomers of **L1** [43,44].

The alkene substrates employed in Rh/**L1** catalysed hydroformylations include terminal alkenes (1-propene and 1-octene), and internal alkenes (isomeric nonenes) [1,38]. As a consequence of their unsymmetrical nature, alkenes other than ethene give linear (*l*) and branched (*b*) aldehydes. For propene, two isomeric aldehydes (one linear and one branched) are formed (reaction i in Scheme 11), while for longer chain alkenes, alkene isomerisation is a competing reaction which can lead to several branched aldehyde products, e.g., for 1-hexene, there are two branched isomers (see reaction ii where R = ^n^Pr in Scheme 11). The *l*:*b* ratio of products is affected by a wide array of factors, including temperature, syngas pressure, ligand–metal (L:Rh) ratio, and the nature of the ligands [7,8,9,25,45]. With monofluorophosphite ligands, it has been shown that the impact of the L:Rh ratio on the alkene hydroformylation activity is strongly dependent on the structure of the ligand. Increasing the L:Rh ratio (L = P-donor ligand) normally decreases catalytic activity, and this is indeed observed with monofluorophosphite **L1**. However, with the bulkier cyclic monofluorophosphite **L16**, increasing the L:Rh ratio increased catalytic activity. The cyclic structure of **L16** appears to be critical for this unusual concentration effect on rate, since the conventional decrease in activity with increase in L:Rh is observed with **L17**, an acyclic analogue of **L16** [46].

A thorough study of the alkene hydroformylation catalytic properties of Rh complexes of monofluorophosphite **L18** has been reported, which includes in-flow and batch hydroformylation of propene, 1-octene, and 2-octene [47]. High activities, with TOF up to 75,000 mol(RCHO) mol(Rh)^−1^ h^−1^, have been observed and outstanding control of the aldehyde *l:b* ratio can be achieved by modulating the temperature, *P*CO, *PH_2_*, time of reaction, the pre-activation of the catalyst, and Rh:**L18** ratio; for example, for 1-octene, the *l:b* ratio can be ‘tuned’ from 0.27 to 15 (corresponding to selectivity ranging from 78% branched to 94% linear). The higher the concentration of **L18**, the more the linear aldehyde is favoured, and this has been rationalised by postulating two mechanisms are operating in parallel: one based on RhL_2_(CO) species, favouring linear aldehyde formation, and the other based on the less bulky RhL(CO)_2_ moiety, favouring branched aldehyde formation [47].

The hydroformylation of ethylene to produce propionaldehyde (Scheme 11, reaction iii) is a potentially useful transformation but acetylene, typically present in ethylene feedstocks in small quantities, acts as a reversible poison towards Rh-based catalysts [25]. The activity of ethylene hydroformylation using a Rh–PPh_3_ catalyst suffered greatly when subjected to ethylene containing 1000 ppm of acetylene. By contrast, the Rh–**L1** catalyst system was shown to be remarkably acetylene-tolerant under the same conditions; the activity of the Rh–**L1** catalyst eventually deteriorated upon increasing the concentration of acetylene to 10,000 ppm [48].

The hydroformylation of formaldehyde (in the form of paraformaldehyde) is potentially a valuable route to produce glycolaldehyde (Scheme 11, reaction iv) which can then be hydrogenated to ethylene glycol. It has been shown that a Rh–**L1** catalyst is more active and selective than a Rh-PPh_3_ catalyst under the same conditions [49].

#### 2.3.2. Other Catalytic Reactions with Monofluorophosphite Ligands

**Scheme 12:**
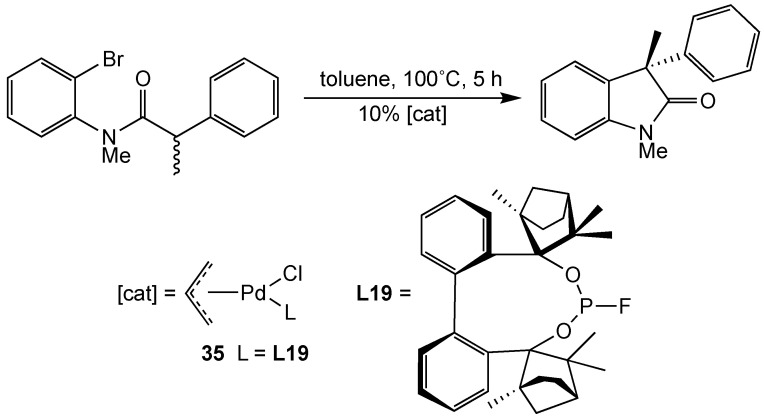
Palladium-BIFOP-F catalysts.

The bulky, optically active monofluorophosphite BIFOP-F (**L19**), derived from fenchol, has been employed in the intramolecular Pd-catalysed cross-coupling reaction shown in Scheme 12 [50]. A library of 12 related fenchol-derived BIFOP-X ligands were screened for catalysis and complex **35,** derived from **L19,** was the most enantioselective (64% *ee*) and gave good yields (88%).

An attempt to use the same ligand **L19** in a Cu-catalysed 1,4-addition of R_2_Zn or RMgBr (R = Me, Et) to enones was unsuccessful; it was suggested that **L19** was unstable under the reaction conditions used [51].

## 3. Monofluorophosphines

### 3.1. Synthesis and Stability of Monofluorophosphines

**Scheme 13:**
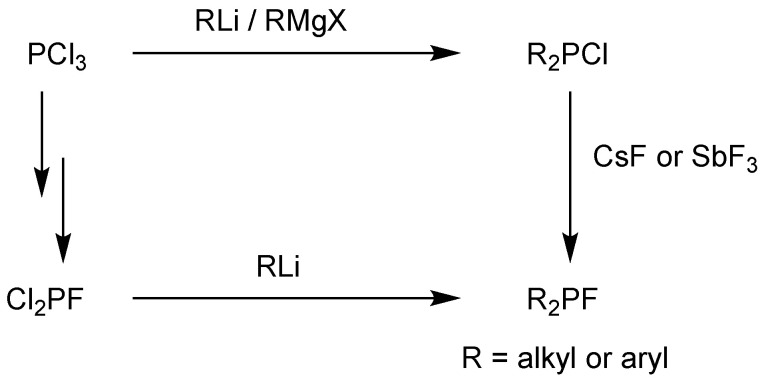
Routes to monofluorophosphines.

Two general routes to R_2_PF where R = alkyl or aryl are shown in Scheme 13. The R_2_PCl route has the advantage of the ready availability of chlorophosphines from PCl_3_ but the Cl_2_PF route can provide access to R_2_PF for which the corresponding R_2_PCl is unknown, as demonstrated for (PhC≡C)_2_PF [52].

**Scheme 14:**
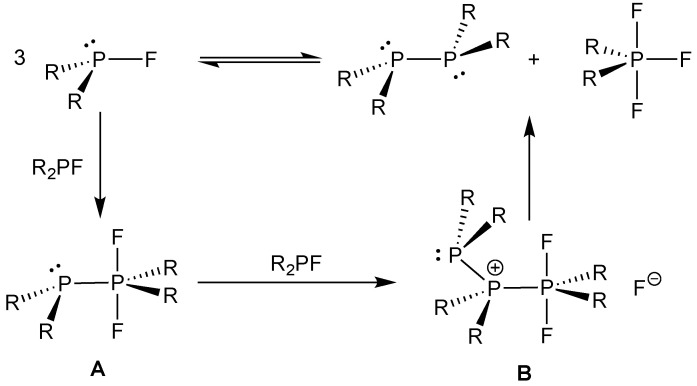
Disproportionation of monofluorophosphines R_2_PF.

Simple R_2_PF (which are P^III^ species) are generally unstable with respect to the disproportionation to the P^V^ in R_2_PF_3_ and P^II^ in R_2_P–PR_2_, as shown in Scheme 14 [53,54]. The pathway shown in Scheme 14, involving the intermediates **A** and **B**, has been proposed for the disproportionation; examples of P^III^–P^V^ species **A** have been isolated and characterised spectroscopically [55,56]. This chemistry would militate against the application of monofluorophosphines as ligands in homogeneous catalysis unless, under the catalytic reaction conditions, the equilibrium in Scheme 14 lies in favour of the R_2_PF, or the equilibrium is rapidly reversible, such that it can be entrained via metal complexation.

The following generalisations on the stability of R_2_PF to disproportionation (Scheme 14) have been established from extensive studies:

(1) Many common R_2_PF (e.g., R = Ph, Me, *^n^*Bu) readily disproportionate [54,57,58];
(2) Bulky substituents and electron-withdrawing substituents stabilise R_2_PF with respect to disproportionation [59,60];
(3) Cyclic monofluorophosphines with constrained C–P–C bonds are more stable with respect to disproportionation than acyclic analogues [61].

**Figure 5:**
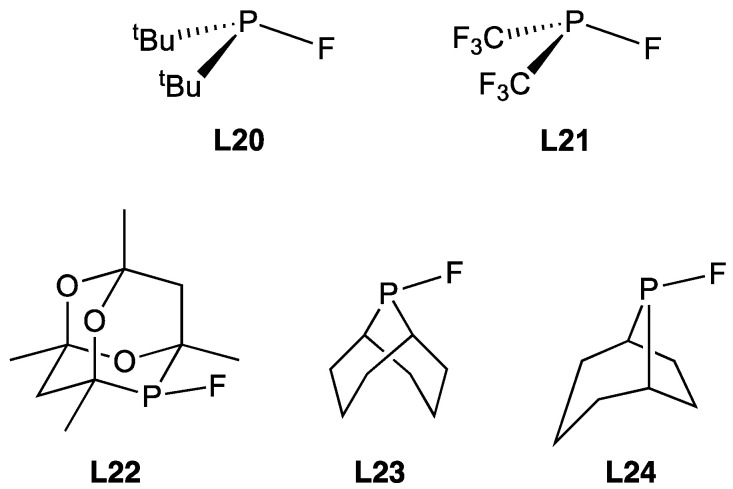
Stable monofluorophosphine ligands.

The stabilising effects of the P-substituents noted in generalisation (2) accounts for the dominance of *^t^*Bu_2_PF (**L20**) and (CF_3_)_2_PF (**L21**) in the early literature concerning the coordination chemistry of monofluorophosphines (Figure 5). A simple rationale for the R_2_PF-stabilising effect of bulky and electron-withdrawing substituents is that these substituents raise the energy of the disproportionation diphosphane product, R_2_P–PR_2_ because (a) bulky R groups maximise 1,2-steric repulsions in the relatively crowded diphosphane—^t^Bu_2_P–P^t^Bu_2_ has been calculated to have a weak P-P bond [62]; (b) electron-withdrawing groups destabilise the P–P bond due to electrostatic repulsion between the resulting δ+ charges on each of the P atoms—it has been reported that (CF_3_)_2_P–P(CF_3_)_2_ has an elongated P-P bond [63]. A mechanism for disproportionation involving sterically crowded intermediates **A** and **B**, which would also be disfavoured by electron-withdrawing substituents [54,55,56], has been proposed (Scheme 14).

The monofluorophosphines CgPF (**L22**), containing a phospha-adamantane cage, and the PhobPF species **L23** and **L24**, containing a phospha-bicycle (Figure 5), are remarkably stable to disproportionation [61]. The CgP and PhobP moieties are rigid and bulky, and so the stability of **L22–L24** may be, at least in part, explained using similar steric congestion arguments to those used above for the stability of **L20** [64,65,66,67]. In addition, it has been argued that the constrained C–P–C angles in **L22**–**L24** also contribute to their observed stability to disproportionation (generalisation (3) above) using the following reasoning [61]. The two geometric isomers of R_2_PF_3_ have diapical–equatorial (*aae*) or apical–diequatorial (*aee*) F groups, with the high apicophilicity of F leading to the *aae* isomer being preferred for R_2_PF_3_ [68]. Therefore, the favoured isomer has the two R substituents occupying two equatorial sites with a 120˚ angle between them, as depicted in Scheme 14. X-ray crystallography has shown that the C–P–C angles are close to 90° in multiple compounds containing either the CgP or PhobP moieties [64,65,66,67]. Consequently, the observed stability to disproportionation of **L22**–**L24** can be partly attributed to the high degree of C–P–C ring strain in R_2_PF_3_ that would be incurred by the 2 C substituents occupying equatorial sites; if, instead, the *eea* isomer were adopted, there would be an unfavourable cost in the P–F bond energies associated with two of the F substituents occupying equatorial sites [68].

### 3.2. Coordination Chemistry of Monofluorophosphines

In general, monofluorophosphine (R_2_PF) complexes are made just like many other P-ligand complexes: by the substitution of a labile ligand on a precursor complex. In metal complexes of monofluorophosphines, the coordinated R_2_PF is not susceptible to disproportionation. Consequently, ligated Ph_2_PF (which is unstable as the free ligand) has been generated within a Cr, Mo, or W coordination sphere by fluoride substitution of a labile X group on a precursor R_2_PX complex [69,70,71].

#### 3.2.1. Group 6 Metal Complexes of Monofluorophosphines

**Scheme 15:**
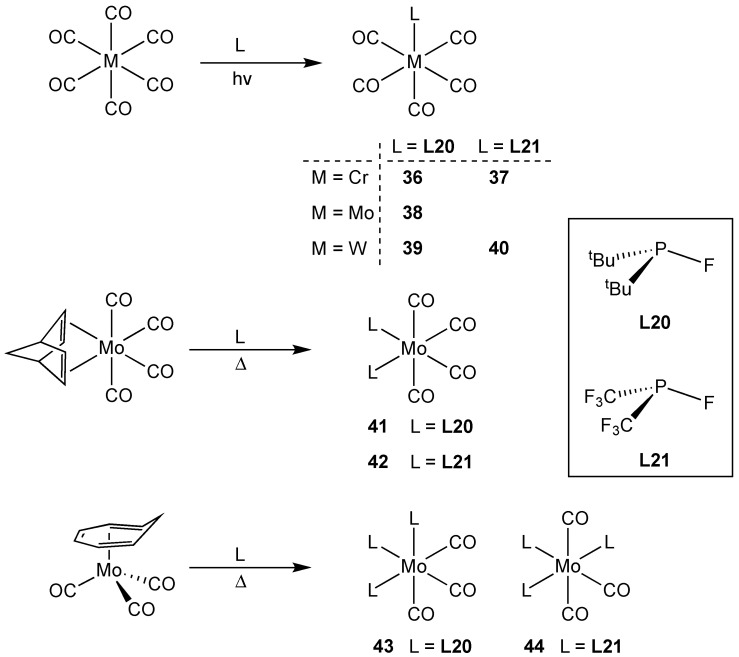
Monofluorophosphine complexes of Group 6 metals.

The Group 6 complexes **36–44** of monofluorophosphines **L20** and **L21** are shown in Scheme 15 [72,73,74]. The [ML(CO)_5_] complexes **36–40** were made by photolysis of a mixture of [M(CO)_6_] and ligand in THF (for **L20**) or CH_2_Cl_2_ (for **L21**) [72,73]. The *cis-*disubstituted complexes **41** and **42** were formed by stirring [Mo(norbornadiene)(CO)_4_] with the ligand at ambient temperatures for several hours [72,74]. The [MoL_3_(CO)_3_] complexes **43** and **44** were both prepared from [Mo(cycloheptatriene)(CO)_3_], but the products were assigned different geometries (*fac* in **43** and *mer* in **44***,* respectively) based on the unambiguous IR and ^19^F NMR spectra for the C_2v_ and C_3v_ isomers. Extensive NMR (^31^P and ^19^F) and IR spectroscopic studies have been carried out on all complexes **36–44**. It was shown that the trend in the position of the highest energy ν_CO_ band in the IR spectra of **36** and its analogues are consistent with the expected π-acidities being in the order: ^t^Bu_3_P (2067 cm^−1^) < ^t^Bu_2_PF (2076 cm^−1^) < ^t^BuPF_2_ (2088 cm^−1^) < PF_3_ (2104 cm^−1^) [74].

**Figure 6:**
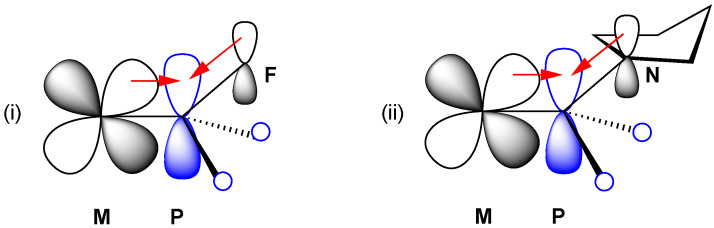
MO pictures of the π-bonding involved in (**i**) fluorophos and (**ii**) pyrrolylphos ligands.

A notable conclusion drawn on the basis of the IR spectra of *cis-*[MoL_2_(CO)_4_] and *mer-*[MoL_3_(CO)_3_] is that (CF_3_)_2_PF and CF_3_PF_2_ are stronger π-acceptors than PF_3_, notwithstanding the greater electronegativity of F than that of CF_3_ (χ of 4.0 and 3.3, respectively, on the Pauling Scale). It has been suggested [74] that an explanation for this apparent anomaly lies in the π component present in the P–F bond that involves a HOMO (lone pair) orbital on F and the LUMO (σ *) on P which has π symmetry. This is the same orbital on P that is involved in the π backbonding from the metal. Thus, in a M–P–F fragment, the M competes with F for the π acceptor orbital on P (Figure 6(i)); this competition is not present in a M–P–CF_3_ fragment which would explain the greater π acceptor capacity of (CF_3_)_2_PF than PF_3_ [74]. This explanation in terms of π interactions between the LUMO (σ *) on P and a HOMO with π symmetry on a P-substituent is reminiscent of the arguments used by Woollins et al. to explain why P*^t^*Bu(pyrrolyl)_2_ is a stronger σ donor than P(pyrrolyl)_3_ (see Figure 6(ii)) [75].

#### 3.2.2. Group 7 Metal Complexes of Monofluorophosphines

**Scheme 16:**
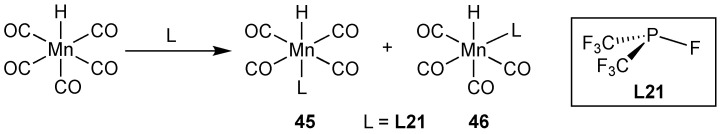
Monofluorophosphine-manganese(I) complexes.

The only reported Group 7 metal complexes containing a monofluorophosphine ligand are the isomeric hydridomanganese(I) complexes **45** and **46**, formed as a 3:1 mixture by the reaction of [HMn(CO)_5_] with **L21** (Scheme 16) [76].The ^2^*J*(HP) values for **46** (72 Hz) and **47** (4 Hz) are consistent with the assignment of their respective *trans* and *cis* geometries.

#### 3.2.3. Group 8 Metal Complexes of Monofluorophosphines

**Scheme 17:**
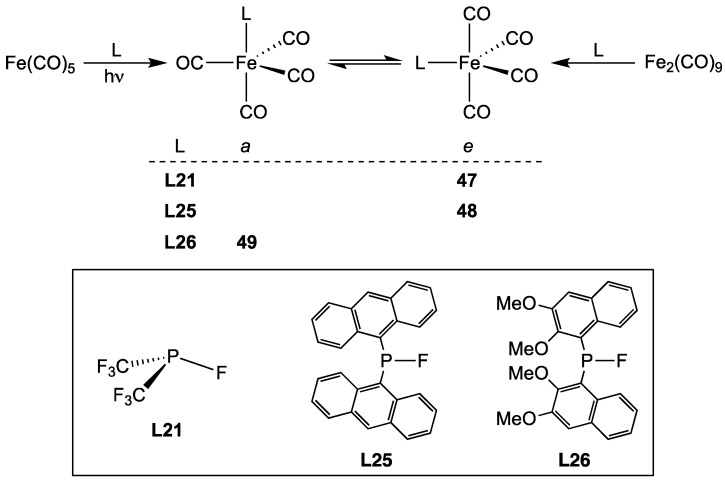
Monofluorophosphine complexes of iron(0).

The tetracarbonyliron complex **47** can be generated in situ by photolysis of a mixture of **L21** and [Fe(CO)_5_] and the IR spectrum suggests that **47** is predominantly the equatorial isomer (Scheme 17). This is consistent with **L21** being bulkier than PF_3_ and of comparable π acceptor capacity to it [29].

The anthracene-derived monofluorophosphine **L25** and the naphthalene-derived monofluorophosphine **L26** were prepared from Cl_2_PF (Scheme 13) [52]. Ligand **L25** was purified by distillation and showed no tendency to undergo disproportionation presumably because it is stabilised by its bulky substituents. Reaction of **L25** with [Fe_2_(CO)_9_] gave complex **48**, whose IR spectrum was consistent with C_2v_ symmetry and was therefore assigned to the equatorial isomer. Ligand **L26** was not obtained in pure form but the impure material was reacted with [Fe_2_(CO)_9_] to produce the iron complex **49**, the IR spectrum of which was consistent with C_3v_ symmetry and was therefore assigned to the apical isomer [52]. The different geometries assigned to **48** and **49** may be rationalised by **L25** being larger and more electron poor (making it a better π-acceptor) than **L26**.

**Scheme 18:**
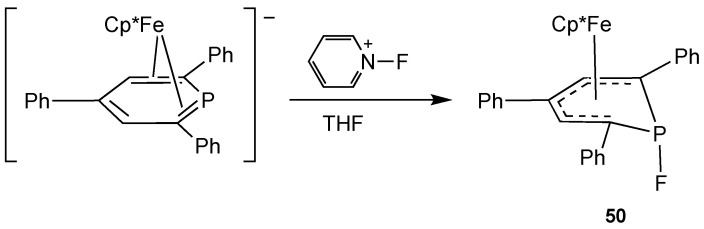
Formation of metalla-monofluorophosphine. Cp* = η^5^-C_5_Me_5_.

The unusual monofluorophosphine **50** has been prepared by treatment of its anionic precursor with *N-*fluoropyridinium tetrafluoroborate which acts as an electrophilic source of F^+^ (see Scheme 18) [77]. The P–F bond in **50** was shown to be covalent in the solid state by single-crystal X-ray diffraction (d_P–F_ = 1.658(4) Å), and in solution by ^31^P and ^19^F NMR spectroscopy, which showed that ^1^*J*_PF_ = 918 Hz). The data for **50** are comparable to values for conventional R_2_PF compounds: d_P–F_ = 1.619(7) Å for ^t^Bu_2_PF [78]; ^1^*J*_PF_ = 905 Hz for Ph_2_PF [57]. In principle, **50** could act as an monofluorophos ligand, but this has not been reported to date.

**Scheme 19:**
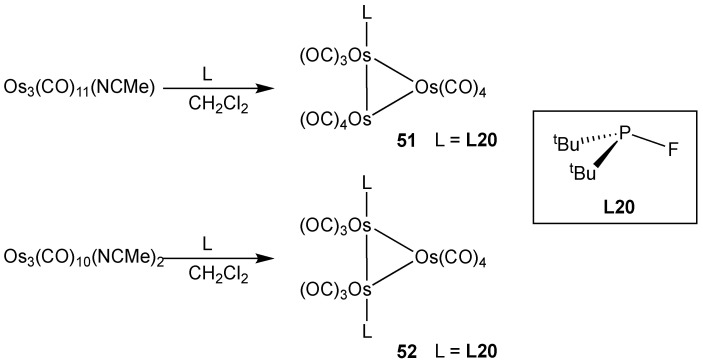
Osmium cluster complexes of monofluorophosphines.

The osmium cluster complexes **51** and **52** were readily formed by the addition of an excess of the bulky monofluorophosphine **L20** to the corresponding labile MeCN complex precursors (Scheme 19) [79].

#### 3.2.4. Group 9 Metal Complexes of Monofluorophosphines

**Scheme 20:**
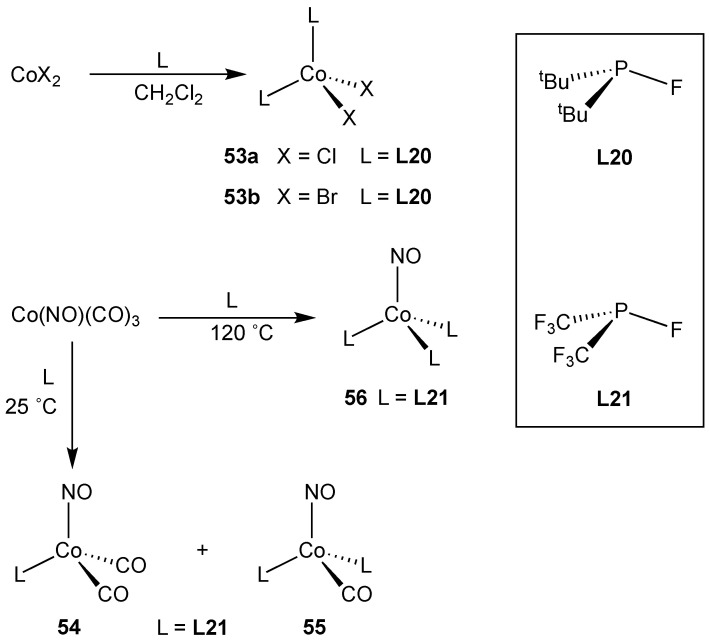
Monofluorophosphine-cobalt complexes.

The paramagnetic cobalt complexes **53a** and **53b** were prepared by stirring a suspension of CoX_2_ in CH_2_Cl_2_ with **L20**. The highly coloured **53a** (blue) and **53b** (blue-green) had electronic spectra, IR spectra, and magnetic moments (μ ≈ 4.5 BM) consistent with the tetrahedral geometry depicted in Scheme 20 [80].

Reaction of 1 equiv. of **L21** with [Co(CO)_3_(NO)] at ambient temperatures over 7 days yielded a mixture of monosubstituted and disubstituted complexes **54** and **55**, which were separated by fractional distillation [81]. The trisubstituted complex **56** was obtained by heating a mixture of [Co(CO)_3_(NO)] and an excess of **L21** to 120 ˚C (Scheme 20). The position of the ν_NO_ band in the IR spectra of **54** (1832 cm^−1^), **55** (1842 cm^−1^), and **56** (1854 cm^−1^) are consistent with **L21** being a better π-acceptor than CO.

**Scheme 21:**
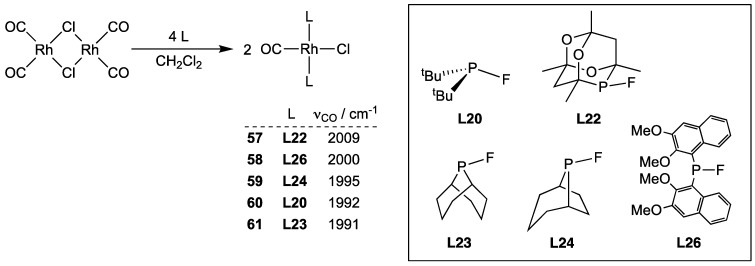
Monofluorophosphine–rhodium complexes.

Mononuclear rhodium complexes **57–61** are formed rapidly upon reaction between [Rh_2_Cl_2_(CO)_4_] and the appropriate monofluorophosphine in CH_2_Cl_2_ (Scheme 21). The v_CO_ values given in Scheme 21 show that the cage monofluorophosphine **L22** is the strongest π-acceptor followed by the dimethoxynaphthalene ligand **L26** and then the *sym* and *asym* isomers of the bicyclic fluorophobanes **L23** and **L24** straddle the bulky **L20** [52,61].

**Scheme 22:**
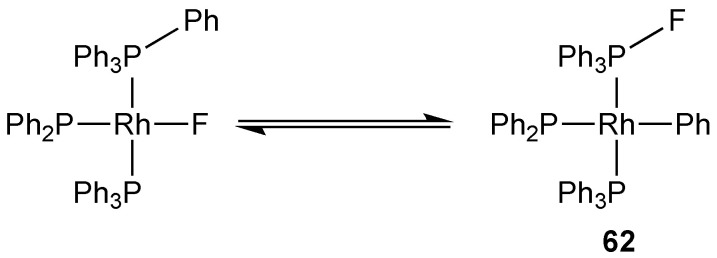
Rearrangement leading to in situ formation of Ph_2_PF complex.

The fluoro analogue of Wilkinson’s Catalyst, [RhF(PPh_3_)_3_], undergoes the rearrangement shown in Scheme 22 to generate complex **62**, which contains a ‘trapped’ Ph_2_P–F ligated to Rh [82]. This remarkable isomerisation occurs under mild conditions and is reversible. Several examples are known where late transition metal fluoro complexes with PR_3_ ancillary ligands undergo related P–C/M–F rearrangements to generate coordinated R_2_P–F ligands as products or transient intermediates [82].

#### 3.2.5. Group 10 Metal Complexes of Monofluorophosphines

**Scheme 23:**
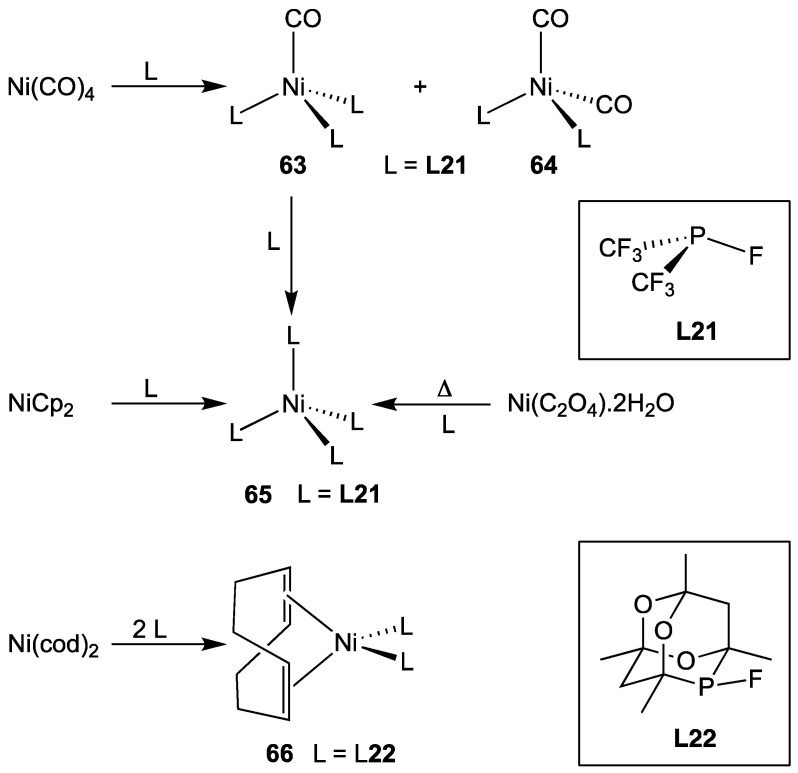
Routes to nickel(0)–monofluorophosphine complexes.

Treatment of nickel tetracarbonyl with an excess of **L21** at 25 °C gives predominantly monocarbonyl **63** with traces of dicarbonyl **64**, which can be separated by fractional distillation. The fully substituted complex **65** is produced under more forcing conditions (95 °C, 24 h), but the product is contaminated with traces of **63** (Scheme 22) [83]. The volatile, air-stable nickel(0) complex **65** can be more readily prepared by mixing **L21** with nickelocene [84] or by reaction of **L21** with metallic nickel, generated by thermolysis of nickel oxalate at 60 °C (Scheme 23) [85]. The reaction between [Ni(cod)_2_] and the phospha-cage flurophosphine **L22** was reported to give complex **66** (Scheme 23), identified in solution on the basis of the stoichiometry used and the characteristic AA’XX’ pattern observed in the ^31^P NMR spectrum [61].

**Scheme 24:**
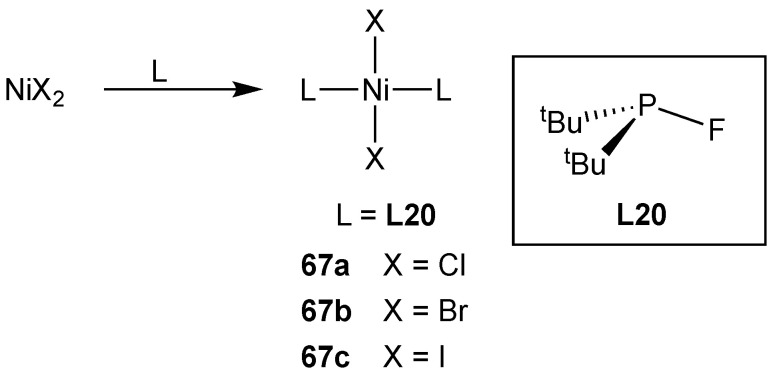
Nickel(II)–monofluorophosphine complexes.

Diamagnetic nickel(II) complexes **67a–c** are formed when suspensions of NiX_2_ in acetone or toluene are treated with **L20** (Scheme 24) [80]. The *trans* geometry of **57a** was established from the large ^2^*J*_PP_ of 425 Hz and the crystal structure of **57b** confirms its *trans* geometry in the solid state [86].

**Scheme 25:**
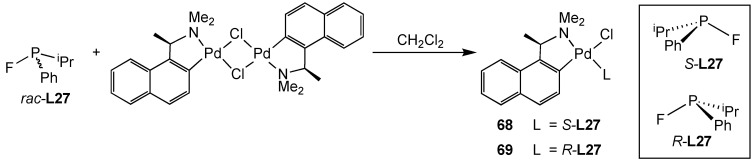
Resolution of optically active monofluorophosphine.

Chiral monofluorophosphine **L27** disproportionates (Scheme 14) over a period of 16 h, but the rate of the disproportionation for dilute solutions of **L27** in benzene was slow enough to measure its optical purity [87]. Reaction of a racemic mixture of **L27** with the optically pure dipalladium complex shown in Scheme 25 gave a diastereomeric mixture of complexes **68** and **69**. Pure complex **68** was obtained selectively by repeated crystallisation from diethyl ether and the absolute configuration at P was determined by X-ray crystallography. Enantiomerically pure *S-***L27** was then displaced from complex **68** by addition of a chelating diphosphine. It was shown by polarimetry that *S-***L27** racemised in benzene over a period of 6 h [87].

**Scheme 26:**
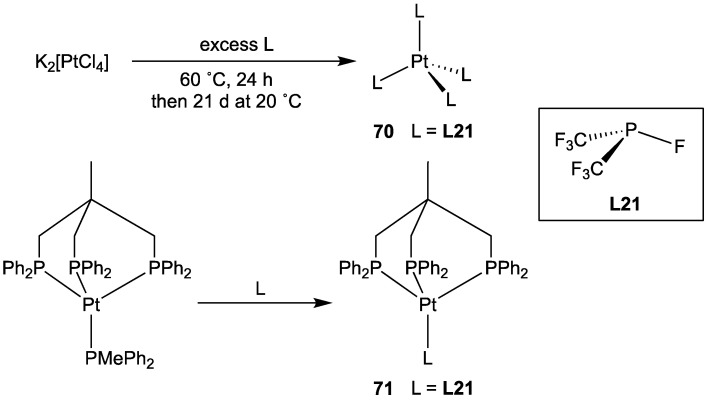
Platinum(0) complexes of monofluorophosphine.

The platinum(0) complex **70** was prepared by heating K_2_[PtCl_4_] (or PtCl_2_) with a large excess of **L21** followed by prolonged shaking at ambient temperature (Scheme 26); the P^V^ by-product (CF_3_)_2_PFCl_2_ was identified, consistent with **L21** acting as the reducing agent [88]. Complex **70** was inert to the addition of MeI, HCl, C_2_H_4_, or CS_2_, even upon prolonged heating, in contrast to the triphenylphosphine analogue [Pt(PPh_3_)_4_]. This behaviour likely reflects the greater π-acceptor properties of **L21** stabilising Pt(0) and reducing its nucleophilicity, coupled with the greater steric bulk of PPh_3_ promoting the formation of reactive, coordinatively unsaturated PtL_3_ species [88].

The insoluble platinum(0) complex **71** was prepared by the replacement of PMePh_2_ by **L21** in the reaction shown in Scheme 26, a reaction presumably driven by the greater π-acceptor properties of **L21** than PMePh_2_ [89].

**Scheme 27:**
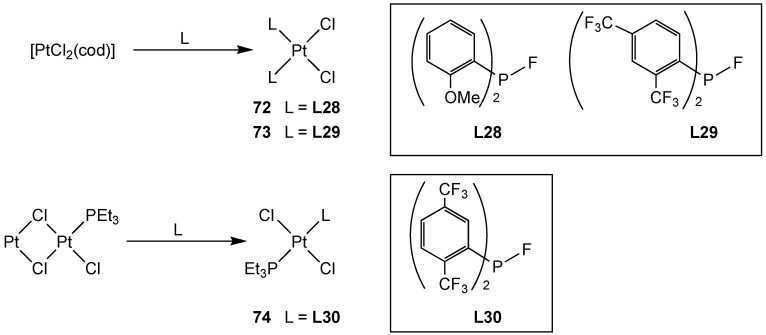
Platinum(II) complexes of monofluorophosphines.

The substituted diarylfluorophosphines **L28**, **L29**, and **L30** form the platinum(II) complexes **72**, **73**, and **74** by the routes shown in Scheme 27. The *cis* geometry of **72** and **73** was confirmed by their X-ray crystal structures [52,90], and the *trans*-configuration of **74** was confirmed by the large value of ^2^*J*_P,P_ = 567 Hz [91].

### 3.3. Catalysis with Complexes of Monofluorophosphines

#### 3.3.1. Hydroformylation Catalysis with Rhodium Complexes of Monofluorophosphines

**Scheme 28:**
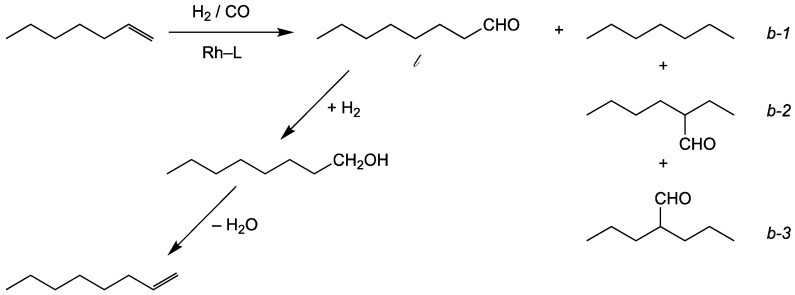
Hydroformylation of 1-heptene.

The first step in the homologation of 1-heptene to 1-octene is the hydroformylation shown in Scheme 28 [92]. Rhodium complexes of monofluorophos ligands **L20**, **L22**, **L23**, and **L24** all showed catalytic activity comparable to the commercialised Rh-–PPh_3_ catalyst. The *l:b* ratio of 3.9 obtained for the Rh–**L22** catalyst compares favourably with the *l:b* ratio of 2.2 for the Rh-PPh_3_ catalyst under the same conditions. The ^31^P NMR spectrum of the exit solutions for the Rh–**L22** catalysis showed the presence of Rh–monofluorophos complexes, indicating that the coordinated **L22** had survived the reaction conditions [61].

#### 3.3.2. Hydrocyanation Catalysis with Nickel Complexes of Monofluorophosphines

**Scheme 29:**
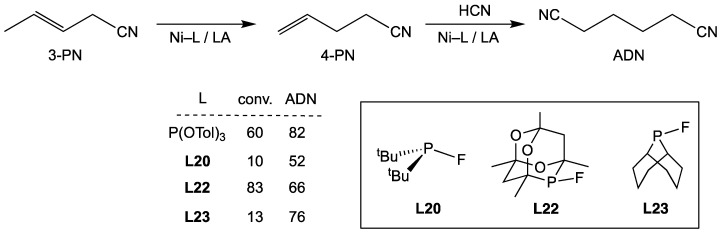
Hydrocyanation to give ADN catalysed by Ni(0)-monofluorophosphine complex.

Catalysts derived from nickel complexes of **L20**, **L22**, **L23,** and **L24** with a Lewis acid (ZnCl_2_ or Ph_2_BOBPh_2_) co-catalyst were tested for the Ni-catalysed isomerisation-hydrocyanation of 3-pentenenitrile (3-PN) to give adiponitrile (ADN) via 4-pentenenitrile (4-PN), as shown in Scheme 29. Nickel complexes of **L24** showed essentially no activity (only traces of ADN detected). Compared with the commercialised catalyst based on Ni–P(OTol)_3_, the Ni–**L20** and Ni–**L23** catalysts were modestly active and selective but Ni–**L22** system showed good activity and selectivity [61,93,94]. The fluorine substituent in CgP–F (**L22**) was critical to the success of the hydrocyanation catalyst (Scheme 29), since attempts to use CgP–Br or CgP–Ph as ligands gave only traces of ADN.

## 4. Conclusions and Prospective Applications of Monofluorophos Ligands in Coordination Chemistry and Catalysis

The combination of the extreme electronegativity and smallness of F has made ligands containing a P–F bond of academic interest for many years. The strength of the P–F bond at 490 kJ mol^−1^ dwarfs other P–X single bonds (*cf*. P–C, 264 kJ mol^−1^; P–O, 335 kJ mol^−1^) and is the source of the thermodynamically stability of P–F compounds. PF_3_ is often characterised as the ultimate π-acceptor, outstripping even CO in its capacity to stabilise electron-rich, low oxidation state metal complexes. What has attracted particular attention to substituted monofluorophos ligands is their capacity to be ‘tunable’ analogues of PF_3_ and indeed to make ligands such as (CF_3_)_2_PF which are more powerful π-acceptors.

The focus of this review has been on the coordination chemistry of monofluorophosphites, (RO)_2_PF, and monofluorophosphines, R_2_PF, and the successful applications of monofluorophos–metal complexes in homogeneous catalysis. At the outset, the prospects for applications of monofluorophos ligands in homogeneous catalysis appeared to be inauspicious because of two fundamental instabilities: (1) notwithstanding the great P–F bond strength, monofluorophos compounds are generally susceptible to hydrolysis, a reaction driven by the formation of the even stronger bonds, H–F (565 kJ mol^−1^) and P=O (544 kJ mol^−1^); (2) the propensity of F to stabilise high oxidation states explains the observation that many P^III^–F compounds readily decompose by disproportionation into P^V^–F compounds and P^II^ species containing P–P bonds.

The 1998 report by Puckette and coworkers at Eastmann of the application of the cyclic monofluorophosphite **L1** in Rh-catalysed hydroformylation under commercially viable conditions and the impressive advantages of this catalyst (including its tunable regioselectivity) emphatically established that monofluorophos ligands have great potential as ligands for catalysis. It was shown that **L1** has structural features that make it resistant to both hydrolysis and disproportionation. These features were borrowed from diphosphites such as **L3** which are: the PO_2_ heterocycle and the bulky hydrophobic t-butyl groups that protect the P–F group and kinetically stabilise the monofluorophosphite.

Early studies (in the 1970s and 1980s) demonstrated that monofluorophosphines **L21** and **L22** were stable to disproportionation and this was rationalised in terms of the great steric bulk and strong electron-withdrawing properties of the substituents. It was later shown that constraining the C–P–C angle in bicyclic or tricyclic monofluorophos ligands such as **L22** also led to greater stability with respect to disproportionation. Ligands such as **L22** have been shown to be effective not only in hydroformylation but also in hydrocyanation under commercially viable conditions.

In view of the observed powerful stabilising effects of P-substituents on monofluorophos ligands, and the demonstrated capacity of monofluorophos ligands to support homogeneous catalysis, it is surprising to us that, to date, the area of monofluorophos chemistry remains so underdeveloped and it is our contention that there are a plethora of opportunities in the areas of ligand design, fundamental coordination chemistry studies, and catalyst discovery based on ligands containing a P–F bond.

It is clear from this review that, firstly, a P–F group confers unusual donor properties on the P^III^ ligand, but there are striking ‘holes’ in our knowledge due to the paucity of information on monofluorophos coordination chemistry of many d-block metals; for instance, to the best of our knowledge, there are no examples of monofluorophos complexes of Re or Au. Secondly, the few catalytic studies on monofluorophos–metal complexes that have been reported have led to impressive discoveries. Some suggestions for potentially fruitful lines of enquiry that build on the results presented in this review are outlined below.

**Figure 7:**
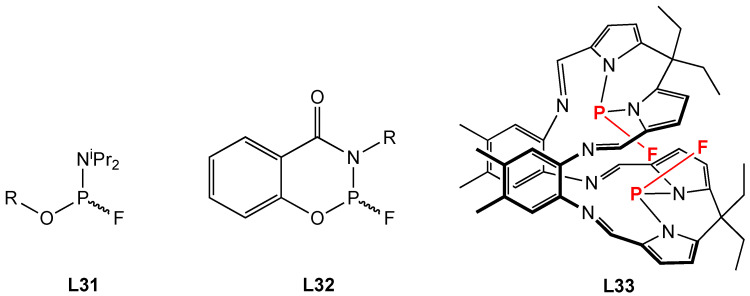
Monofluorophos ligands worthy of future study for catalysis.

The monofluorophosphites, denoted {O,O}PF, and monofluorophosphines, denoted {C,C}PF, that are the subject of this review represent only a minor portion of the monofluorophos landscape that is available (Figure 2). There are many related {N,N}PF as well as mixed {C,O}PF, {C,N}PF, and {N,O}PF ligands waiting to be developed. Indeed, a series of acyclic and cyclic {N,O}PF ligands, (see Figure 7) of general structure **L31** (R = alkyl) [95] and **L32** (R = aryl or alkyl) [96], have been reported. Ligand **L32** generates Rh catalysts for alkene hydroformylation with *l:b* ratios ranging from 0.41 to 12.8 depending on ligand concentration and the nature of R [96].

Chelating bis(monofluorophos) ligands would be an exciting avenue to explore and an example of a bis{N,N}PF ligand was recently described: the “Pacman” fluorophos ligand **L33** (see Figure 7) [97].

Hydroformylation and hydrocyanation catalysis have been successfully demonstrated with monofluorophos ligands. These observations are consistent with the monofluorophos ligands behaving like other P-donors that are relatively electron-poor, such as phosphites. Monofluorophos–metal catalysts should be capable of catalysing other reactions that are catalysed by metal-phosphites and related ligands such as alkene isomerisation, hydrogenation, and C-C coupling reactions.

It was discovered that the optically active monofluorophosphite **L19** was an effective ligand for the enantioselective Pd-catalysed intramolecular C–C coupling reaction. It would certainly be of interest to develop other optically active monofluorophos ligands (including bidentates) and investigate their efficacy in asymmetric catalysis. All of the {X,Y}PF heterocycles shown in Figure 2 have a stereogenic P-centre, and it should be possible to resolve these molecules and investigate the application of their complexes in asymmetric catalysis.

The overarching conclusion is that there is great scope to design new fluorophos ligands containing a PF group and expand the range of steric and electronic effects such ligands can have. There are good reasons to believe that new catalysts will emerge.

## Data Availability

Not applicable.
